# Hypoxia suppresses cylindromatosis (CYLD) expression to promote inflammation in glioblastoma: possible link to acquired resistance to anti-VEGF therapy

**DOI:** 10.18632/oncotarget.2216

**Published:** 2014-07-14

**Authors:** Jianying Guo, Satoru Shinriki, Yu Su, Takuya Nakamura, Mitsuhiro Hayashi, Yukimoto Tsuda, Yoshitaka Murakami, Masayoshi Tasaki, Takuichiro Hide, Tatsuya Takezaki, Jun-ichi Kuratsu, Satoshi Yamashita, Mitsuharu Ueda, Jian-Dong Li, Yukio Ando, Hirofumi Jono

**Affiliations:** ^1^ Department of Diagnostic Medicine, Graduate School of Medical Sciences, Kumamoto University, Kumamoto, Japan; ^2^ Department of Clinical Pharmaceutical Sciences, Graduate School of Pharmaceutical Sciences, Kumamoto University, Kumamoto, Japan; ^3^ Department of Pharmacy, Kumamoto University Hospital, Kumamoto, Japan; ^4^ Department of Neurology, Graduate School of Medical Sciences, Kumamoto University, Kumamoto, Japan; ^5^ Department of Oral and Maxillofacial Surgery, Graduate School of Medical Sciences, Kumamoto University, Kumamoto, Japan; ^6^ Department of Breast and Endocrine Surgery, Graduate School of Medical Sciences, Kumamoto University, Kumamoto, Japan; ^7^ School of Medicine, Kumamoto University, Kumamoto, Japan; ^8^ Department of Neurosurgery, Graduate School of Medical Sciences, Kumamoto University, Kumamoto, Japan; ^9^ Center for Inflammation, Immunity and Infection and Department of Biology, Georgia State University, Atlanta, Georgia

**Keywords:** bevacizumab, CYLD, glioblastoma, hypoxia, inflammation

## Abstract

Cylindromatosis (CYLD) is a tumor suppressor that regulates signaling pathways by acting as a deubiquitinating enzyme. CYLDdown-regulation occurred in several malignancies, with tumor-promoting effects. Although we found loss of CYLD expression in hypoxic regions of human glioblastoma multiforme (GBM), the most aggressive brain tumor, biological roles of CYLD in GBM remain unknown. This study aimed to determine the biological significance of CYLD down-regulation to GBM progression and therapy. CYLD mRNA transcription was dramatically down-regulated in hypoxic GBM cells, consistent with our clinical observations of human GBM tissues. Hypoxia enhanced both basal and tumor necrosis factor-α-induced expression of various proinflammatory cytokines, whereas CYLD overexpression strongly counteracted these responses. In addition, chronic anti-angiogenic therapy with bevacizumab, an anti-vascular endothelial growth factor (VEGF) antibody, with enhanced hypoxia produced responses similar to these CYLD-regulated proinflammatory responses in a xenograft mouse model. Histologically, CYLD clearly prevented massive immune cell infiltration surrounding necrotic regions, and pseudopalisades appeared in bevacizumab-treated control tumors. Furthermore, CYLD overexpression, which had no impact on survival by itself, significantly improved the prosurvival effect of bevacizumab. These data suggest that CYLD down-regulation is crucial for hypoxia-mediated inflammation in GBM, which may affect the long-term efficacy of anti-VEGF therapy.

## INTRODUCTION

Glioblastoma multiforme (GBM), the most common primary malignant brain tumor, has features of rapid and invasive growth in the brain [1]. The median overall survival of patients who have standard and targeted therapies is still just more than 1 year, mostly because of resistance to therapy [2]. Because few therapeutic targets are available for GBM, better understanding of the molecular mechanisms of GBM progression and therapy resistance is important.

Hypoxia, a characteristic of malignant tumors, frequently outpaces their blood supply [3]. Hypoxic regions often occur in GBM, and increased tumor hypoxia is associated with the resistance to chemotherapy and radiation and the poor prognosis of GBM patients [4]. The hypoxic microenvironment promotes invasion and treatment resistance of GBM cells, and glioma-initiating cells possess strong drug resistance and tumorigenicity [4, 5]. Pseudopalisading cells commonly surround hypoxia-induced necrotic areas, a configuration that is unique to GBM and has been recognized for some time as a feature indicating poor prognosis [6]. Of note, several recent publications have discussed the clinical significance of hypoxia-elicited inflammation [7]. The inflammatory microenvironment, the seventh hallmark of cancer, generally promotes malignant progression [8]. Indeed, various inflammatory cytokines promote the growth, survival, and invasion of GBM cells [9-11]. Increased expression of inflammatory cytokines including angiogenic mediators such as vascular endothelial growth factor (VEGF) has been associated with the poor prognosis of GBM [9-13]. Hypoxia promoted the activation of nuclear factor-κB (NF-κB), a primary mediator of inflammatory responses, and was involved in regulating the inflammatory microenvironment [7, 8, 14-17]. However, little is still known about molecular mechanisms linking hypoxia and inflammation and the effects of this relationship on GBM progression and treatment resistance.

In addition to the hypoxic microenvironment being clinically significant for GBM, vascularization is critical: GBM is one of the most highly vascularized tumors and expresses high levels of VEGF, which is therefore an attractive target for anti-angiogenic therapies [18]. Bevacizumab, a humanized monoclonal antibody against VEGF, is approved for recurrent and newly diagnosed GBM. Clinical trials with bevacizumab produced impressive radiographic responses and prolongation of progression-free survival. However, GBM inevitably progresses within months [19], and the impact of this therapy on overall survival is not clear [20, 21]. Recent evidence has indicated that prolonged anti-angiogenic treatment leads to tumors developing progressive hypoxia, which is thought to be important for resistance to therapy, in view of basal tumor-promoting roles of hypoxia in GBM [22, 23]. However, the effects of hypoxia induced by anti-angiogenic treatment on tumor progression during therapy and ultimately on overall survival in GBM patients remain uncertain.

Cylindromatosis (CYLD) was first identified in familial cylindromatosis as a mutant tumor suppressor gene [24]. CYLD is a deubiquitination enzyme targeting lysine 63-linked ubiquitin chains and was shown to negatively regulate signal transduction factors and pathways including transforming growth factor-β and NF-κB signaling pathways [25-27]. CYLD regulates diverse biological processes including cell proliferation, survival, and migration; immune responses; osteoclastogenesis; and spermatogenesis [26]. With regard to malignancies, reduced expression and mutation of CYLD, with tumor-promoting effects, were reported for several cancers including melanoma, T-cell leukemia, hepatocellular carcinoma, and breast cancer [28-31]. CYLD expression was recently shown to be reduced in gliomas, with an inverse correlation with tumor grade and prognosis [32]. However, mechanisms underlying CYLD down-regulation and involvement of CYLD in pathological processes crucial for GBM progression, including hypoxia response and treatment resistance, remain largely unknown.

In our study here, we found CYLD down-regulation in hypoxic regions of human GBM tissues, with CYLD acting as a critical regulator of hypoxia-mediated inflammation in GBM, which may be associated with resistance to anti-VEGF therapy. These findings may allow insights into the role of CYLD in the pathogenesis of GBM and may contribute to improve the survival benefit of anti-VEGF therapy in GBM.

## RESULTS

### Hypoxia-induced CYLD Down-regulation May Be Associated with the Inflammatory Microenvironment in GBM

**Figure 1:**
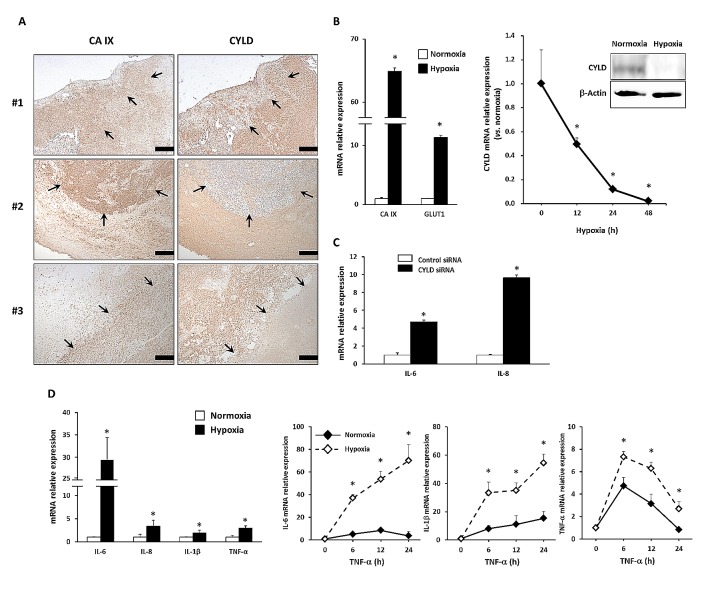
Effects of hypoxia on expression of CYLD and proinflammatory cytokines in GBM cells (A) Immunohistochemical analysis with anti-CYLD and anti-CA IX antibodies in GBM tissues. In representative photomicrographs from 3 GBM cases, arrows indicate areas with high CA IX and low CYLD expression. Scale bars indicate 500 μm. (B) U87MG cells were incubated under hypoxic conditions, after which mRNA expression of hypoxic markers (left panel) and CYLD mRNA (right panel) were determined via qPCR. CYLD protein expression was determined by using Western blotting 24 h after hypoxic culture (right panel). * P< 0.05. (C) Expression of IL-6 and IL-8 mRNA was determined via qPCR after CYLD knockdown by using siRNA. * P< 0.01. (D) mRNA expression of inflammatory cytokines (left panel) was determined with qPCR under normoxic and hypoxic conditions. Cells were incubated with 10 ng/mL TNF-α for the indicated periods under normoxic and hypoxic conditions, and then mRNA expression of IL-6, IL-1β, and TNF-α was determined by using qPCR (right panels). * P< 0.05 compared with normoxic conditions. Values are means ± SE of triplicate samples.

To investigate CYLD expression in GBM, we first performed immunohistochemical analysis with anti-CYLD antibody in human GBM tissues (n = 48). As Figure 1A shows, some GBM cells appeared to be positive for CYLD immunoreactivity. Our additional comparison with expression of carbonic anhydrase IX (CA IX), a well-known marker of hypoxia, clearly showed a loss of CYLD immunoreactivity in hypoxic regions, defined as showing a high CA IX expression (Fig. 1A). To determine whether hypoxia influences CYLD expression, we cultured U87MG cells under hypoxic conditions of 1% O_2_. We confirmed an increased expression of the hypoxia markers CA IX and glucose transporter 1 (GLUT1) (Fig. 1B *left panel*). Consistent with our clinical observations, CYLD mRNA expression was dramatically reduced in U87MG cells under hypoxic conditions in a time-dependent manner (Fig. 1B *right panel*). After 48 h of hypoxic culture, the CYLD mRNA level significantly decreased to only 2.3% of that of normoxic culture. We also confirmed reduced CYLD expression at the protein level (Fig. 1B *right panel*), which suggests involvement of CYLD in certain hypoxia-related processes in GBM.

Some publications have discussed hypoxia-elicited inflammation [7, 14, 15], and a loss of CYLD has been associated with overproduction of inflammatory cytokines and various immune processes [25, 26]. Indeed, CYLD knockdown by using small interfering RNA (siRNA) significantly increased expression of the proinflammatory cytokines interleukin (IL)-6 and IL-8 in GBM cells (Fig. 1C). We also found that hypoxia significantly increased expression of typical proinflammatory cytokines including IL-6, IL-8, IL-1β, and tumor necrosis factor (TNF)- α in GBM cells (Fig. 1D *left panel*). Moreover, hypoxia strongly enhanced TNF-α-triggered induction of IL-6, IL-1β, and TNF-α compared with normoxia (Fig. 1D *right panels*), which indicates increased sensitivity of hypoxic GBM cells to inflammatory stimuli. Together, these findings suggest that hypoxia-induced CYLD reduction may be involved in inflammatory responses in GBM.

### CYLD Inhibited Hypoxia-induced Inflammatory Responses

**Figure 2:**
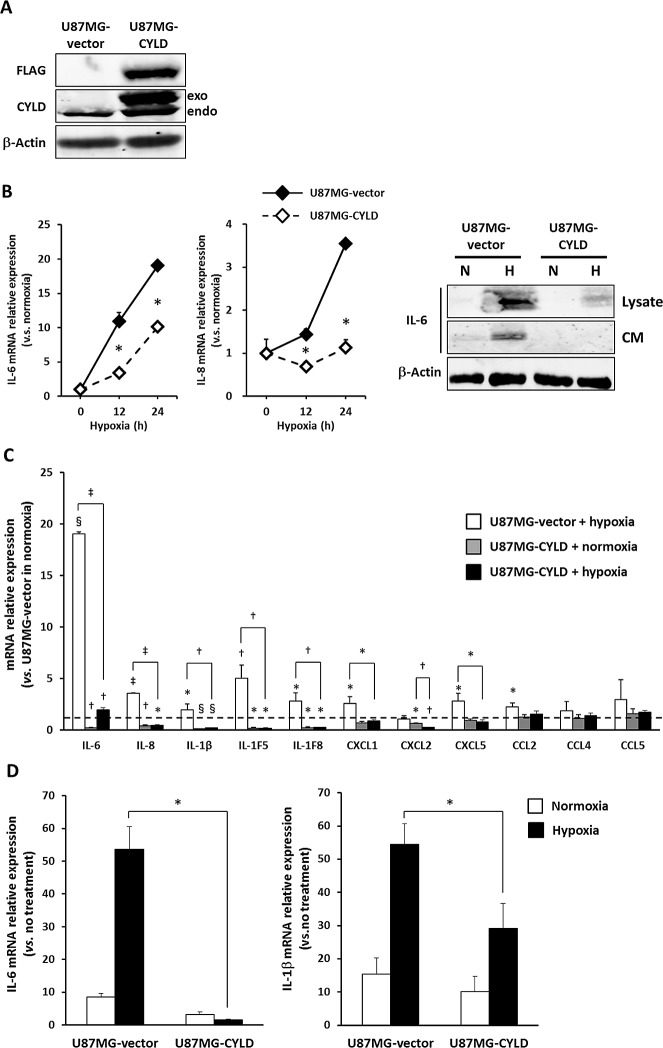
Overexpression of CYLD inhibited hypoxia-induced inflammatory responses (A) Western blotting to determine the relative amounts of FLAG-tagged exogenous (exo) CYLD protein versus endogenous (endo) protein. (B) Each stable cell line was incubated for the indicated periods under hypoxic conditions, after which mRNA expression of IL-6 (left panel) and IL-8 (middle panel) was determined via qPCR. * P< 0.05 compared with normoxic conditions. Values are means ± SE of triplicate samples. IL-6 protein level (right panel) in total cell lysates (Lysate) and conditioned medium (CM) under normoxic (N) and hypoxic (H) conditions was determined via Western blotting. (C) mRNA expression of various inflammatory cytokines in U87MG-vector and U87MG-CYLD cells under normoxic or hypoxic conditions was determined via qPCR. The dashed line indicates the expression level of each cytokine in U87MG-vector cells under normoxic conditions. * P< 0.05, † P< 0.01, § P< 0.001, and ‡ P< 0.0001 compared with U87MG-vector cells in normoxia unless otherwise indicated. (D) Cells were incubated with 10 ng/mL TNF-α for 2 h under normoxic or hypoxic conditions, and mRNA expression of IL-6 (left panel) and IL-1β (right panel) was determined via qPCR. The Y-axis shows the fold change in mRNA expression versus no treatment. * P< 0.0005. Values are means ± SE of triplicate samples.

To determine whether a substantial reduction in CYLD expression played a vital role in induction of proinflammatory cytokines and an excessive response to inflammatory stimuli under hypoxic conditions, we established a stable U87MG cell line overexpressing wild-type CYLD (U87MG-CYLD) and a control line (U87MG-vector) (Fig. 2A). In fact, CYLD overexpression significantly inhibited hypoxia-triggered induction of IL-6 and IL-8 (Fig. 2B *left panel*). CYLD overexpression clearly inhibited hypoxia-induced synthesis and secretion of IL-6 (Fig. 2B *right panel*).

On the basis of these results, we studied other inflammatory mediators. In addition to expression of IL-6, IL-8, and IL-1β, expression of interleukins IL-1F5 and IL-1F8 and of chemokines CXCL1, CXCL5, and CCL2 increased significantly during hypoxia (Fig. 2C). In fact, CYLD overexpression completely blocked hypoxia-mediated induction of those inflammatory mediators and suppressed basal expression of some cytokines (IL-6, IL-8, IL1β, IL-1F5, IL-1F8, and CXCL2) (Fig. 2C). CYLD overexpression also significantly suppressed a synergistic increase in IL-6 or IL-1β expression induced by TNF-α stimulation in hypoxia (Fig. 2D). These results indicated that a hypoxia-induced CYLD decrease contributed to induction of inflammatory mediators and increase in sensitivity to inflammatory stimuli under hypoxic conditions in GBM cells.

### CYLD Blocked Infiltration of Inflammatory Cells and Invasion Around Anti-VEGF Therapy-enhanced Hypoxic Regions in GBM Xenografts

**Figure 3:**
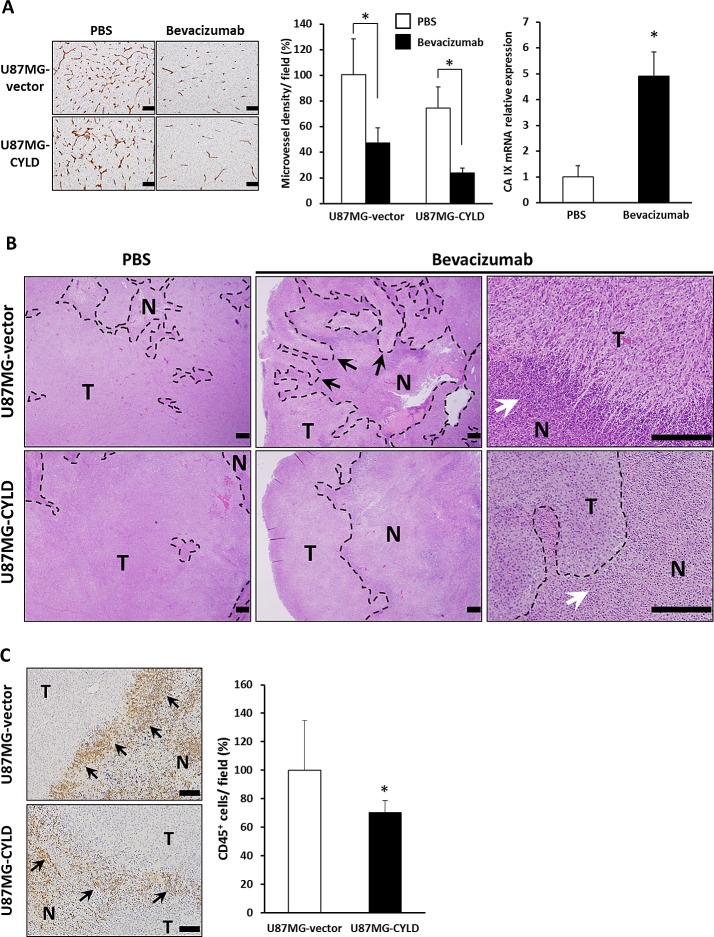
Effects of chronic bevacizumab treatment on histology of GBM xenografts (A) Sections of U87MG-vector and U87MG-CYLD tumors treated with PBS or bevacizumab were stained with an antibody against the endothelial cell surface marker CD31 (left panel). Scale bars indicate 400 μm. Microvessel density was quantified by morphometric analysis (middle panel). Values are the average of 3 to 4 independent tumors per experimental condition. CA IX mRNA expression in U87MG-vector tumors after 18 days of PBS or bevacizumab treatment was determined via qPCR (right panel). * P< 0.01. (B) Histological H&E staining analysis of tumor tissues after 18 days of PBS or bevacizumab treatment. Photomicrographs show representative examples of tumor sections in each experimental condition. N and T indicate necrotic and tumor areas, respectively. Black and white arrows indicate an invasive front and infiltration of inflammatory cells in necrotic margins, respectively. The upper right panel shows pseudopalisades. Scale bars indicate 500 μm. (C) Immunohistochemical analysis with an antibody against the inflammatory cell surface maker CD45 (left panels). CD45+ cells were quantified (right panel). Values are the average of 3 to 4 independent tumors per experimental condition. Scale bars indicate 100 μm. * P< 0.05.

We next sought to validate our in vitro findings in an in vivo model. Given that angiogenesis is essential for O_2_ supply and nutrition during tumor growth [3, 4], we utilized anti-angiogenic therapy with bevacizumab, an anti-VEGF antibody used for GBM, to enhance hypoxia in GBM xenografts. We found no quantitative difference in microvessel density and tumor growth between U87MG-vector and U87MG-CYLD xenografts (Fig. 3A *left* and *middle panels*, and data not shown). Bevacizumab treatment had almost the same inhibitory effect on vascularization (U87MG-vector, 53%; U87MG-CYLD, 51%) and tumor growth in both groups (Fig. 3A *left* and *middle panels*, and data not shown). These results indicated that high levels of CYLD in GBM cells had little impact on angiogenesis and the anti-angiogenic efficacy of bevacizumab. We also confirmed that bevacizumab treatment significantly increased CA IX mRNA expression (Fig. 3A *right panel*), which indicates an increase in hypoxic regions. Our histological examinations thus clearly indicated that necrotic areas increased in both tumors (Fig. 3B *left* and *middle panels*). Of note, we observed wide invasion fronts surrounding necrotic areas (Fig. 3B *upper middle panel*) and, at higher magnification, frequent perinecrotic pseudopalisades, a configuration that indicates migrating hypoxic GBM cells [6], with massive infiltration of inflammatory cells in bevacizumab-treated tumors (Fig. 3B *upper right panel*). Particularly notable findings included CYLD-overexpressing tumors having a smoother hypoxic margin consisting of round tumor cells even after treatment (Fig. 3B *lower middle* and *right panels*). In addition, infiltration of CD45^+^ inflammatory cells surrounding hypoxic margin in CYLD-overexpressing tumors after treatment was significantly fewer than control tumors (Fig. 3C). Together, these findings suggest that CYLD restricts infiltration of inflammatory cells and invasion enhanced by increased hypoxia after anti-VEGF therapy in GBM.

### CYLD Improved Survival of Bevacizumab-treated GBM-bearing Mice via Suppression of Inflammatory Cytokines

**Figure 4:**
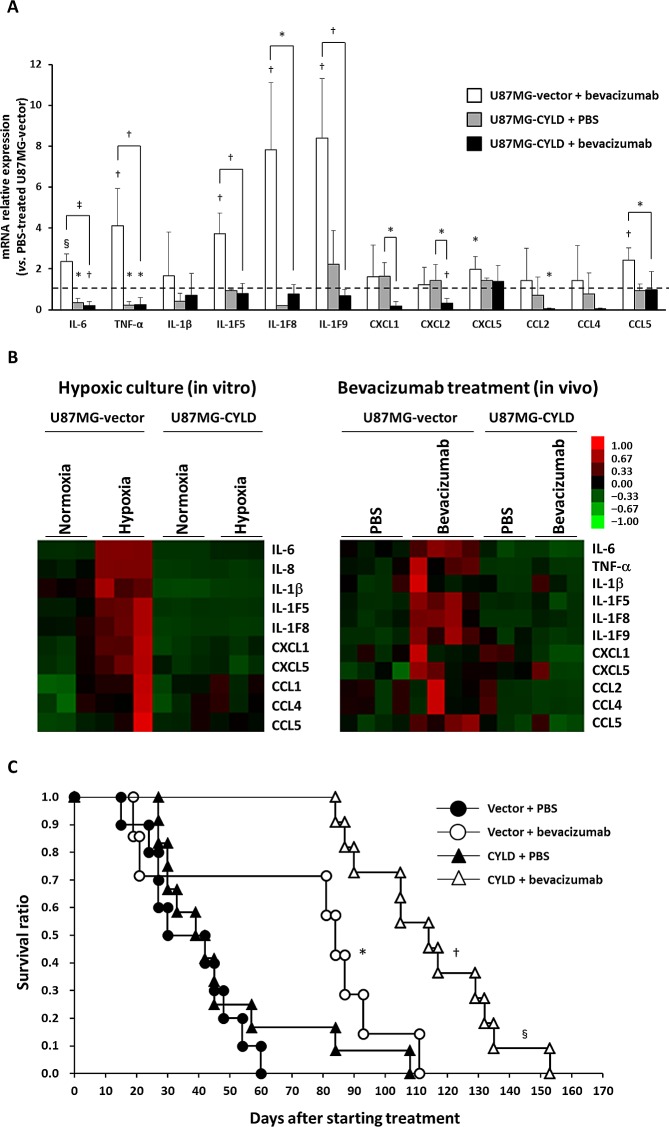
Effects of CYLD overexpression on expression of inflammatory cytokines and survival of mice after bevacizumab treatment (A) mRNA expression of various inflammatory cytokines in U87MG-vector and U87MG-CYLD tumors treated with PBR or bevacizumab for 18 days was determined via qPCR. The dashed line indicates the expression level of each cytokine in U87MG-vector tumors treated with PBS. * P< 0.05, † P< 0.01, § P< 0.001, and ‡ P< 0.0001 compared with 87MG-vector tumors treated with PBS unless otherwise designated. (B) Heat maps representing gene expression changes observed in in vitro (left panel) and in vivo (right panel) experiments. (C) Kaplan-Meier plots of overall survival in each experimental group. * P< 0.05 compared with the PBS-treated U87MG-vector group; † P< 0.0001 compared with the PBS-treated U87MG-CYLD group; § P< 0.005 compared with the bevacizumab-treated U87MG-vector group (log-rank test).

We next investigated expression of proinflammatory cytokines and determined the involvement of CYLD in long-term efficacy of bevacizumab in the GBM xenograft model. Consistent with the results of hypoxic culture in vitro, the expression of several proinflammatory cytokines, including TNF-α, IL-6, IL-1 families (IL-1F5, IL-1F8, and IL-1F9), CXCL5, and CCL5, increased significantly in human U87MG-vector cells in mouse xenografts after chronic bevacizumab treatment (Fig. 4A). CYLD overexpression suppressed expression of these cytokines to the same or lower level than that in untreated tumors. Expression of certain cytokines such as CXCL1, CXCL2, and CCL2, which were not apparently induced by treatment, was also significantly down-regulated by CYLD overexpression after treatment. Our analysis using heat maps showed more clearly the similarities in expression patterns of different cytokines in in vitro hypoxic culture and after in vivo bevacizumab treatment (Fig. 4B), which indicates that CYLD indeed acted as a negative regulator of hypoxia-mediated inflammation.

We also investigated whether CYLD overexpression in GBM cells affected survival of mice with or without bevacizumab treatment. As Figure 4C illustrates, the effect of CYLD overexpression in GBM cells on survival was not significant. In fact, although continued bevacizumab treatment significantly prolonged survival of both groups of mice (U87MG-vector, P = 0.0138; U87MG-CYLD, P< 0.0001, log-rank test), the survival time of treated mice bearing CYLD-overexpressing tumors was significantly longer than that of treated mice bearing control tumors (median survival 12.0 weeks vs 16.3 weeks, P = 0.0028, log-rank test). These findings suggest that CYLD improves the survival of bevacizumab-treated GBM-bearing mice via inhibition of therapy-induced inflammatory responses.

## DISCUSSION

Hypoxia is a significant biological phenomenon in various solid tumors. Given the well-established clinical relationship between increased hypoxia and GBM *progression* [4, 5, 33], targeting *hypoxia*-induced processes may be essential for developing successful treatment of G*BM*. The most important finding from this study is that hypoxia-induced CYLD reduction may be critical for inflammatory responses triggered by hypoxia. In a recent report, gliomas had reduced CYLD expression that was inversely correlated with tumor grade and prognosis [32]. In the present study, our thorough assessment of clinical samples and in vitro analysis revealed that CYLD expression was reduced under hypoxic conditions via transcriptional regulation. As of today, little is still known about transcriptional regulation of CYLD. However, CYLD mRNA transcription is directly inhibited by Snail [28] and the Notch target Hes1 [29], both of which are up-regulated and activated under hypoxic conditions [34, 35]. Another finding worth noting is that hypoxia stimulates human papilloma virus-encoded E6 protein to promote ubiquitination and proteasomal degradation of CYLD in human papilloma virus-positive squamous cell carcinoma cell lines [36]. With regard to downstream CYLD signals, mounting evidence that CYLD negatively regulates NF-κB activity [8, 16] substantially supports our findings. Because TNF-α rapidly activates the NF-κB pathway [8], an excessive inflammatory response to the cytokine under hypoxic conditions may be due largely to CYLD down-regulation. It is interesting that our data showed, for the first time to our knowledge, inhibition by CYLD of the expression of several cytokines in a hypoxia-specific manner, which indicated the presence of hypoxia-specific molecular mechanisms regulated by CYLD. Although additional studies are needed to clarify the molecular mechanisms, our in vitro data suggested that hypoxia-induced CYLD reduction may promote inflammation in an autocrine and paracrine fashion in GBM tissues.

Chronic anti-angiogenic therapy induces hypoxia, which is thought to be crucial for resistance to such therapy [22, 23]. In the present study, we found that chronic administration of bevacizumab, a monoclonal anti-VEGF antibody used for GBM, induced expression of proinflammatory cytokines with massive infiltration of immune cells in GBM xenografts. One important finding was that CYLD overexpression in GBM cells not only prevented those proinflammatory responses but also significantly improved the prosurvival effect of bevacizumab, which by itself had no impact on survival. Although many clinical trials of bevacizumab showed prolonged progression-free survival, the impact of this therapy on overall survival remains limited in GBM and other cancers [19-21], which reflects multiple mechanisms of adaptation to this treatment. Consistent with this result, because no apparent difference in basal vascularity, tumor growth, and anti-angiogenic efficacy itself of bevacizumab between the two tumor groups was observed, the prosurvival effect of CYLD overexpression may depend on modulation of phenotypic alterations occurring during bevacizumab treatment. Growing evidence exists that anti-VEGF treatment has antitumor effects but simultaneously induces tumor adaptation and progression to greater malignancy, with increased invasiveness [19, 37]. Indeed, we clearly found pseudopalisades, a characteristic feature of GBM that is currently thought to indicate tumor cells' actively migrating away from central hypoxic areas [6, 38, 39].

Among high-grade gliomas, the mesenchymal type, characterized as having gene expression associated with invasion, is in the class with the worst prognosis, with treatment resistance [40, 41]. Several clinical and preclinical studies with U87MG cells demonstrated that mesenchymal transition of GBM cells during chronic anti-VEGF treatment underlies a diffuse relapse [42-45]. The predominant biological process occurring during the transition was an inflammatory response [43, 44]. Also, TNF-α converted GBM cells to a mesenchymal subtype [11]. In addition, increased infiltration of myeloid cells reflected recurrence after anti-angiogenic therapy [44, 46, 47]. Such an increased myeloid cell influx correlated strongly with the degree of tumor hypoxia [44]. Moreover, hypoxic GBM cells including pseudopalisading cells [6] highly expressed the mesenchymal marker c-Met [45].

Thus, the increased invasiveness of GBM cells during anti-VEGF therapy is likely due to an enhanced inflammatory milieu associated with hypoxia induction. Together, our data suggest that inhibition of the GBM cell-derived inflammatory response by CYLD overexpression led to less aggressiveness, including invasion, and ultimately better survival although orthotopic xenograft model for exact prognostic evaluation remains to be examined. The concept that regulation of inflammatory processes is important during anti-VEGF therapies may be supported by a recent finding that indirect activation of helper T17 cells by bevacizumab was crucial for acquired resistance to the therapy [48]. Continued investigation should contribute to clarification of mechanisms underlying resistance to anti-angiogenic therapies that inevitably facilitate hypoxia, and development of efficient combination therapy based on “action-reaction” theory [49].

In conclusion, we demonstrated that CYLD acts as a critical regulator of hypoxia-mediated inflammation in GBM, which may affect the long-term efficacy of anti-VEGF therapy. Deeper understanding of the mechanisms linking hypoxia-induced CYLD down-regulation and inflammation, and adaptive changes in GBM tissues during anti-VEGF therapy, may provide insights into GBM pathobiology and development of more effective therapeutic approaches to GBM.

## METHODS

### Antibodies and Reagents

Rabbit polyclonal anti-CA IX antibody was obtained from Novus Biologicals (Littleton, CO, USA). Rabbit polyclonal anti-CYLD antibody for immunoblotting and rabbit polyclonal anti-CYLD antibody for immunohistochemistry were purchased from Santa Cruz Biotechnology (Santa Cruz, CA, USA) and Sigma (St. Louis, MO, USA), respectively. Rat monoclonal anti-mouse CD45 antibody was also obtained from Santa Cruz Biotechnology. Goat polyclonal anti-human IL-6 antibody and anti-mouse CD31/PECAM-1 antibodies were obtained from R&D Systems (Minneapolis, MN, USA). Mouse monoclonal anti-FLAG antibody was purchased from Sigma. pcDNA3-FLAG-CYLD and the empty plasmids were kindly provided by Dr. Jian-Dong Li.

### Patients and Tissue Specimens

GBM tissue specimens were obtained from 48 patients with GBM who underwent resection at the Department of Neurosurgery, Kumamoto University Hospital, between 2008 and 2011. The average age of the patients (± SD) was 63 ± 15.8 years; 24 men and 24 women were enrolled. Samples were used for immunohistochemical analysis. This study followed the guidelines of the Ethical Committee of Kumamoto University. We explained the nature and aims of the study to all subjects, who gave informed consent for participation.

### Cell Culture

The human GBM cell line U87MG, kindly provided by Dr. Takuichiro Hide, Department of Neurosurgery, Kumamoto University, was authenticated via short tandem repeat fingerprinting by the Japanese Collection of Research Bioresources Cell Bank. Cells were grown in Dulbecco's modified Eagle's medium and Ham's F-12 medium (Invitrogen, Life Technologies, Carlsbad, CA, USA), with 10% fetal bovine serum, in 5% CO_2_ at 37°C unless otherwise stated. To establish stable control and CYLD-overexpressing U87MG cell lines, either an empty vector or the wild-type CYLD expression plasmid was transfected by using Lipofectamine 2000 (Invitrogen, Life Technologies) according to the manufacturer's protocol, and cells were selected with G418 (300 μg/mL).

### Transfection with siRNA

Cells were transfected with CYLD-specific siRNA by using Lipofectamine 2000 (Invitrogen, Life Technologies) according to the manufacturer's protocol. Silencer Negative Control siRNA (Applied Biosystems, Life Technologies, Foster City, CA, USA) was used as the control. CYLD-specific siRNA sequences were sense 5′- GAUUGUUACUUCUAUCAAAtt-3′ and antisense 5′- UUUGAUAGAAGUAACAAUCtt-3′ (Applied Biosystems, Life Technologies).

### Hypoxia Induction

*In vitro* experiments were performed in a temperature- and humidity-controlled hypoxic chamber set at 1% O_2_, 5% CO_2_, and 94% N_2_ (CO_2_ multigas incubator AMP-30D; ASTEC, Fukuoka, Japan). In some cases, cells were incubated under normoxic or hypoxic conditions for 48 h and were treated with 10 ng/mL TNF-α for the indicated periods.

### Tumor Xenograft Generation and Bevacizumab Treatment

Male CB17/ICR-scid/scid mice (SCID mice), each 8 weeks old and weighing 20–25 g, were obtained from CLEA Japan (Tokyo, Japan) and maintained in a specific pathogen-free environment at the Center for Animal Resources and Development of Kumamoto University. All animal experiments were reviewed and approved by the Kumamoto University Ethics Committee for Animal Experiments (authorization number in Kumamoto University: C23-319, C24-212). U87MG-vector and U87MG-CYLD cells were trypsinized, washed with serum-free Dulbecco's modified Eagle's medium and Ham's F-12 medium, and resuspended in phosphate-buffered saline (PBS), after which their concentration was adjusted to 2 × 10^6^ cells/100 μL in PBS. Cell suspensions were then injected subcutaneously into SCID mice. Tumor development was followed in individual animals by sequential caliper measurements of length (L) and width (W). Tumor volume was calculated by the formula LW^2^π/6. When the average tumor volume was 500 mm^3^, each mouse (n = 7–12/group) received intraperitoneal injections of 100 μL of PBS containing bevacizumab (Avastin; Genentech, Roche, Basel, Switzerland; 5 mg/kg) or PBS alone every 3 days. For survival experiments, treatment continued until the mice died. For tumor analyses, mice (n = 3 or 4/group) were killed on day 18 after treatments started and tumors were removed. Pieces of tumor tissues were sharply excised, placed in sterile tubes, and immediately frozen in liquid nitrogen. All tissue samples for quantitative PCR (qPCR) were stored at −80°C until analysis. For immunohistochemical and hematoxylin-eosin (H&E) staining, tumor tissues were fixed immediately in 10% neutral buffered formalin.

### Histology and Immunohistochemistry

Formalin-fixed specimens of clinical tissues and excised tumor tissues from SCID mice were embedded in paraffin, cut into 4-μm-thick sections, and mounted on slides. These paraffin-embedded sections were dewaxed in xylene and rehydrated in graded alcohols. For immunohistochemistry, sections were incubated with proteinase K (Dako, Glostrup, Denmark) for 15 min at room temperature. Endogenous peroxidase was blocked by incubating slides with 3% hydrogen peroxide for 30 min. After slides were washed with PBS, nonspecific background staining was blocked by using nonspecific staining blocking reagent (Dako) for 15 min, followed by overnight incubation at 4°C with anti-human CYLD antibody (1:200), anti-human CA IX antibody (1:1000), anti-mouse CD45 antibody (1:50), or anti-mouse CD31 antibody (1:50) diluted in PBS containing 1% bovine serum albumin. After slides were rinsed with PBS, they were incubated for 1 h with horseradish peroxidase-conjugated secondary antibodies. Chromogen was developed with 3,3-diaminobenzidine (Dako). All slides were lightly counterstained with hematoxylin. For H&E staining, sections were stained in hematoxylin for 1 min and eosin for 30 s.

The number of CD45^+^ cells was determined in 10 fields per section at ×200 in areas identified as “hot spots” at ×40 surrounding necrotic areas. Results were expressed as an average of the total number of CD45^+^ cells in each field.

### RNA Isolation and qPCR

Total RNA was isolated from tissue specimens and treated cells by using the RNeasy Mini Kit (Qiagen, Valencia, CA, USA) and was reverse transcribed to cDNA by using the ExScript RT reagent kit (Takara Bio Inc., Otsu, Japan), according to the manufacturers' protocols. All PCR reactions were performed via the LightCycler System (Roche Diagnostics, Basel, Switzerland) with SYBR Premix DimerEraser (Takara Bio Inc.). Primers used for qPCR were as follows: CYLD forward: 5'-TCAGGCTTATGGAGCCAAGAA-3', reverse: 5'-ACTTCCCTTCGGTACTTTAAGGA-3'; 18S rRNA, forward: 5'-CGGCTACCACATCCAAGGAA-3', reverse: 5'-GCTGGAATTACCGCGGCT-3'. Primers for inflammatory cytokines were purchased from RealTimePrimers, LLC (Elkins Park, PA, USA) and Sigma. 18S rRNA was used as an internal control.

### Protein Extraction and Immunoblotting

Cells were washed once in ice-cold PBS and then lysed by adding CelLytic M Cell Lysis/Extraction Reagent (Sigma) containing freshly added protease inhibitor cocktail (Sigma), 50 mM NaF, and 1 mM Na_3_VO_4_. Supernatants were stored at −80°C until use. Equal amounts of protein were fractionated via sodium dodecyl sulfate−polyacrylamide gel electrophoresis and transferred to nitrocellulose membranes (GE Healthcare, Little Chalfont, UK). Membranes were blocked with 5% non-fat dried milk and 0.1% Tween 20 (Sigma) in PBS (pH 7.4) and were then incubated overnight at 4°C with antibodies against IL-6 (1:2000), CYLD (1:600), FLAG (1:3800), or β-actin (1:5000) in 5% bovine serum albumin (Sigma) and 0.1% Tween 20 in PBS (pH 7.4). After the membranes were washed in 0.1% Tween 20 in PBS (pH 7.4), they were incubated for 1 h in horseradish peroxidase-conjugated secondary antibodies for 1 h. After another washing, specific protein bands were detected by using ECL Prime Western Blotting Detection Reagents (Amersham Life Science, Arlington Heights, IL, USA), according to the manufacturer's instructions.

### Statistical Analysis

Student's *t*-test was used to assess differences between experimental groups. All statistical analyses were performed by using JMP software Version 5.1 for Windows (SAS Institute Japan, Tokyo, Japan). Statistical significance was defined as P< 0.05.
